# Hydrogel-based co-culture and application of two types of islet cells

**DOI:** 10.1371/journal.pone.0347425

**Published:** 2026-04-28

**Authors:** Lingyan Chen, Miaomiao Zhu, Xinliang Liu, Qiong Wei

**Affiliations:** 1 Department of Endocrinology, Zhongda Hospital, School of Medicine, Southeast University, Nanjing, China; 2 Huaian Maternal and Child Health Care Hospital Affiliated to Yangzhou University Medical College, Huaian, Jiangsu, China; 3 Department of Endocrinology, People’s Hospital of Xuyi, Jiangsu, China; 4 Department of Endocrinology, Zhongda Hospital, Southeast University, Nanjing, China; Advanced Materials Technology Research Institute, National Research Centre, EGYPT

## Abstract

This study aimed to develop a hydrogel-based co-culture system for pancreatic α- and β-cells to mimic native islet composition and achieve bidirectional blood glucose regulation. Monodisperse sodium alginate microsphere encapsulating α-TC6 and β-TC6 cells in a 2:8 ratio were fabricated using a microfluidic electrostatic spray platform. Key processing parameters (voltage, collection distance, flow rate, and alginate concentration) were optimized to precisely control microsphere diameter, yielding highly uniform spheres with excellent monodispersity. The alginate hydrogel exhibited favorable swelling properties and viscoelasticity, providing a supportive 3D microenvironment. In vitro, the microspheres demonstrated high biocompatibility, with cell viability exceeding 95% after 72 hours of co-culture. Encapsulation did not impair cellular function, as evidenced by unhindered insulin and glucagon secretion compared to unencapsulated controls, in streptozotocin-induced diabetic C57BL/6 mice, transplantation of these cell-laden microspheres into the subcutaneous brown fat significantly improved glucose homeostasis. Treated mice showed markedly better glucose tolerance during intraperitoneal glucose tolerance tests and maintained lower fasting blood glucose levels compared to sham-operated and unencapsulated cell transplantation groups. Furthermore, the treatment alleviated diabetes-associated weight loss, with the microsphere group showing a significant weight increase post-transplantation. Histological analysis confirmed the biocompatibility of the implants, with no significant pathological changes in major organs. In conclusion, this sodium alginate microsphere system effectively co-cultures functional islet cells, provides immunoisolation, and restores bidirectional glucose regulation in a diabetic mouse model, offering a promising strategy for pancreatic islet modeling and cell- based diabetes therapy.

## Introduction

Diabetes mellitus (DM) encompasses a group of metabolic disorders characterized by hyperglycemia, arising from either inadequate insulin secretion or insulin resistance. According to the International Diabetes Federation, approximately 537 million adults worldwide were living with diabetes in 2021, and this number is projected to rise to 783 million by 2045 [1]. Type 1 diabetes (T1DM), an autoimmune destruction of pancreatic β-cells, remains a particular therapeutic challenge [2,3]. While exogenous insulin is lifesaving, its inability to achieve precise physiological glycemic control leads to risks of hypoglycemia and long-term complications [4–9].

Pancreatic islets are mini-organs containing endocrine cells that orchestrate glucose homeostasis. Insulin-secreting β-cells (≈70% of islet cells) lower blood glucose, while glucagon-secreting α-cells (≈20%) elevate it. This delicate balance is lost in T1DM. Islet transplantation has emerged as a promising therapy to restore endogenous regulation [10]. However, its widespread application is hampered by critical limitations: chronic graft rejection requiring lifelong immunosuppression, acute inflammatory responses post-transplantation, and a severe shortage of donor organs [11].

To overcome these hurdles, encapsulation of islets within biocompatible hydrogels, such as sodium alginate, has been extensively explored [12–15]. Despite progress, current encapsulation strategies often focus on β-cells alone, neglecting the crucial role of α-cells in physiological counter-regulation. Furthermore, achieving uniform, monodisperse microcapsules with optimal physicochemical properties (e.g., swelling, mechanical strength) using scalable fabrication techniques remains a significant challenge [16–21]. Recent advances in material science, such as zwitterionic modification of alginate, have shown promise in mitigating foreign body responses [22–24], yet the intefration of a co-culture system within a precisely engineered delivery vehicle is underexplored.

**Fig 1:**
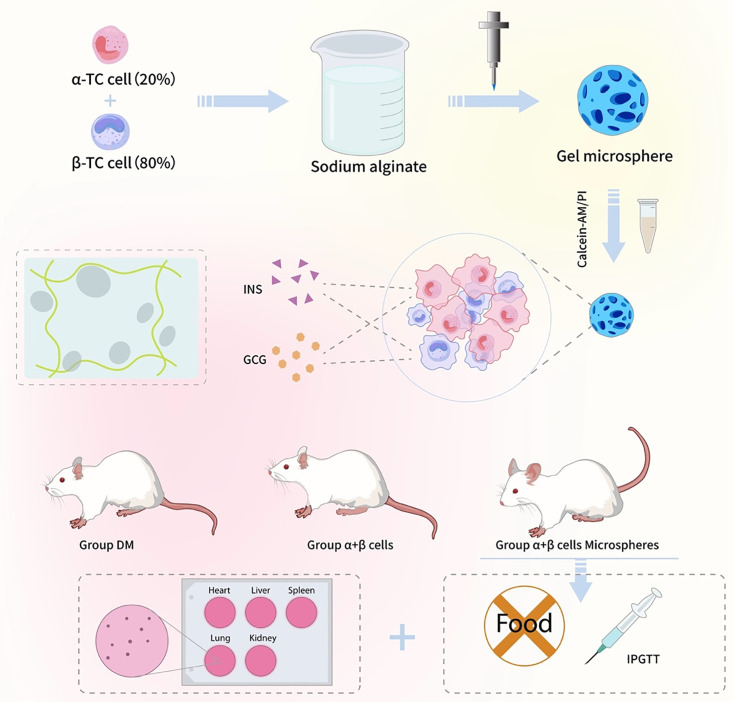
Schematic illustration of fabrication of hydrogel-based microspheres encapsulating islet cells for diabetes treatment after omentum transplantation in diabetic mice.

Therefore, to advance towards a more physiological and durable cell-based therapy for T1DM, key gaps must be addressed: (i) recapitulating the native islet cellular composition (α/β-cell cross-talk) within the graft, (ii) developing a reproducible fabrication method for uniform, bicompatible microcarriers, and (iii) providing robust experimental validation of both in vitro funcion and in vivo therapeutic efficacy. To bridge these gaps, this study presents a comprehensive strategy focusing on three interconnected innovations. First, we designed and fabricatd monodisperse sodium alginate microspheres using a custom microfluidic electrostatic spray platform, allowing precise control over micosphere size and morphology through tunable parameters (voltage, flow rate, collection distance). Second, we established a functional co-culture system within these microspheres, encapsulating pancreatic α-TC6 and β-TC6 cells in a ratio (2:8) mimicking human islet composition. Finally, we rigorously evaluated this construct, demonstrating its excellent biocompatibility, unimpaired hormone secretion in vitro, and, most importantly, its capability to restore bidirectional blood glucose regulation and ameliorate diabetic symptoms in streptozotocin-induced diabetic mice. This work not only provides a promising platform for islet replacement therapy but also serves as a robust and physiologically relevant in vitro model for diavetes research. The overall experimental design and transplantation strategy is schematically depicted in Fig 1.

## Materials and methods

### Materials and platform preparation

All animal experimental procedures received prior approval from the Animal Investigation Ethics Committee of Southeast University (Approval No. 20240223023) and were conducted in accordance with the NIH Guide for the Care and Use of Laboratory Animals. Mice were housed under SPF conditions with a 12-hour light/dark cycle and provided with autoclaved food and water ad libitum.

Sodium alginate (Sigma-Aldrich) was used as the primary hydrogel material. Calcium chloride (CaCl₂) and other chemical reagents were sourced from Aladdin. Cell culture media and supplements were obtained from HyClone and ThermoFisher. Six- to eight-week-old male C57BL/6 mice were purchased from Shanghai Sipo-Bikai Laboratory Animal Co., Ltd.

The core microfluidic device was a coaxial capillary chip. Briefly, a square glass capillary (inner dimension: 1.05 mm) was fixed on a glass slide. A blunt 22-gauge stainless steel needle, serving as the inner phase inlet, was centrally aligned and secured within the square capillary using quick-curing epoxy resin.

The electrostatic spray platform was assembled by integrating this chip with peripheral systems. A syringe pump was connected to the inner needle to dispense the alginate solution. A high-voltage DC power supply (5–15 kV) was connected to the metallic needle. A grounded petri dish containing 2% (w/v) CaCl₂ gelling solution was positioned 5–15 cm below the capillary tip as the collection bath.

### Material characterization

The morphology of the fabricated alginate microspheres was examined by scanning electron microscopy (SEM). Samples were fixed, dehydrated through an ethanol gradient, and subjected to critical-point drying. The dried microspheres were sputter-coated with a 10-nm gold-palladium layer and imaged using a field-emission SEM operated at an accelerating voltage of 5 kV.

The swelling behavior of the hydrogel was quantified by immersing freeze-dried microspheres of known dry weight (W₀) in phosphate-buffered saline (PBS, pH 7.4) at 37°C for 48 hours. The surface liquid was removed with filter paper, and the swollen weight (Wₓ) was recorded to calculate the equilibrium swelling ratio (Q = [(Wₓ – W₀)/ W₀] × 100%). Rheological properties were measured using a rotational rheometer with a 20-mm parallel-plate geometry. Disc-shaped hydrogel samples (1 mm thickness) were subjected to oscillatory frequency sweeps from 0.1 to 10 Hz at a constant strain of 0.2% and temperatures of 25°C and 37°C to obtain the storage modulus (G′) and loss modulus (G″).

### In vitro cell experiments

Cell viability was assessed using an MTT assay. α-TC6 and β-TC6 cells, co-cultured in a 2:8 ratio, were seeded at 5 × 10³ cells per well in 96-well plates and incubated for 24, 48, and 72 hours. Following incubation, 20 μL of MTT solution (5 mg/mL) was added to each well. After 4 hours, formazan crystals were dissolved in DMSO, and absorbance was measured at 490 nm.

Live/Dead staining was performed to visually confirm biocompatibility. Cells were incubated with a solution containing 2 μM Calcein-AM and 4.5 μM propidium iodide (PI) for 30 minutes at 37°C. Fluorescence microscopy was then used to distinguish live (green) from dead (red) cells, and viability was quantified using ImageJ software.

The glucose-stimulated insulin secretion (GSIS) function was evaluated. Cells or microspheres were pre-incubated in low-glucose Krebs-Ringer Bicarbonate (KRB) buffer (2.8 mM) for 1 hour, then stimulated with high-glucose KRB buffer (20 mM) for 1 hour. The supernatant was collected, and insulin concentration was quantified using a mouse insulin ELISA kit.

Glucagon secretion capacity was assessed under low-glucose stimulation. The co-culture systems were first equilibrated in standard medium (6 mM glucose) and then stimulated with low-glucose KRB buffer (1 mM) for 1 hour. The secreted glucagon in the supernatant was measured using a glucagon radioimmunoassay kit.

### In vivo animal study

type 1 diabetes model was established in male C57BL/6 mice via a single intraperitoneal injection of streptozotocin (STZ, 150 mg/kg) dissolved in 0.1 M citrate buffer (pH 4.4). Mice with persistent non-fasting blood glucose levels >16.5 mmol/L one week post-injection were considered diabetic and used for subsequent studies.

Diabetic mice were randomly assigned to three groups (n = 6 per group): a sham-operated group (DM), a group receiving transplantation of free α and β cells (α+βcells), and a treatment group receiving alginate microsphere-encapsulated α/β cells (α + βMicrocapsules). All grafts contained a total of 2 × 10⁶ cells at a 2:8 α/β ratio. Under isoflurane anesthesia, a dorsal incision was made, and the graft was implanted into the subcutaneous brown adipose tissue depot. The incision was closed in layers, and postoperative analgesia (ketoprofen, 5 mg/kg/day, s.c.) was administered for three consecutive days.

Metabolic monitoring was performed weekly. Non-fasting blood glucose and body weight were recorded. An intraperitoneal glucose tolerance test (IPGTT) was conducted at week 2 post-transplantation after a 6-hour fast. Mice were injected with glucose (2 g/kg body weight), and blood glucose levels were measured via tail-vein sampling at 0, 15, 30, 60, 90, and 120 minutes.

At the experimental endpoint (4 weeks post-transplantation), mice were euthanized via an overdose of anesthetic. Blood samples were collected for serum analysis. Major organs (heart, liver, spleen, lungs, kidneys) and the graft site were harvested, fixed in neutral-buffered formalin, and processed for paraffin embedding.

Tissue sections (5 μm thickness) were stained with hematoxylin and eosin (H&E) following standard deparaffinization and rehydration protocols. Stained sections were examined under a light microscope for histopathological assessment.

### Statistics analysis

Data processing was performed using SPSS version 20.0, a widely utilized software for the analysis of various datasets. Measurements that followed a normal distribution were expressed as mean ± standard deviation (SD) to represent both central tendency and variability. Independent sample t-tests were conducted to evaluate mean differences between groups, a crucial method for determining statistical significance. Furthermore, Original version 9.0 and GraphPad Prism version 5 were employed for graphical and analytical representation. A significance threshold of P < 0.05 was established to indicate statistical significance, reflecting the likelihood of observing the data under the null hypothesis and suggesting a reliable experimental outcome when this threshold is surpassed. However, all analyzed data supporting the results and conclusions presented in this manuscript are either contained within the article or provided in the supplementary data file (S1 Data). The statistical analyses were performed on these final datasets.

## Results

### Design and Assembly of a Microfluidic Electrostatic Spray Platform for Microsphere Generation

**Fig 2:**
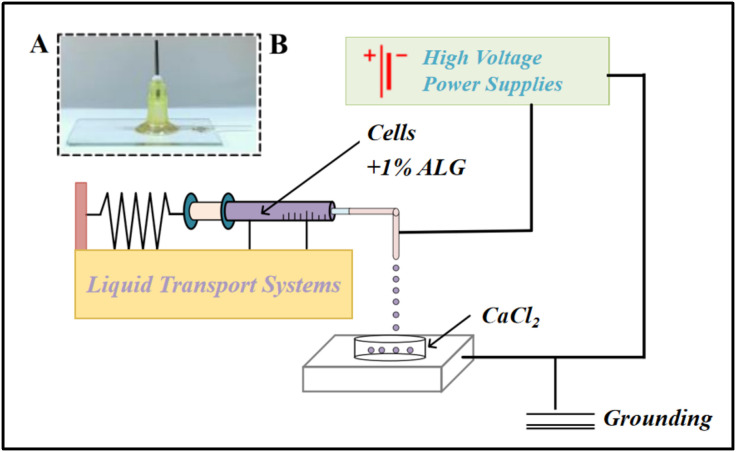
Illustration of the custom microfluidic setup for electrospray, showing the chip and peripheral instruments.

The cornerstone of our fabrication strategy was a custom-built microfluidic electrostatic spray platform engineered for the consistent generation of monodisperse alginate microspheres (Fig 2A). This system integrated a glass capillary-based microfluidic chip (Fig 2B) with a precision syringe pump for controlled fluid delivery and a high-voltage DC power supply. The alginatesolution, upon exiting the chip tip, was atomized into uniform droplets under the applied electric field and subsequently gelled into solid microspheres in a calcium chloride collection bath. This integrated setup provided independent and precise control over key processing variables governing microsphere formation, including electric field strength, flow rate, and collection distance.

### Precise Control of Microsphere Diameter Through Processing Parameters

To quantitatively establish the relationship between processing conditions and microsphere geometry, we systematically investigated the individual effects of applied voltage, collection distance, flow rate, and alginate concentration on microsphere diameter. All experiments, unless specified otherwise, were conducted using a 2% (w/v) sodium alginate solution.

**Fig 3:**
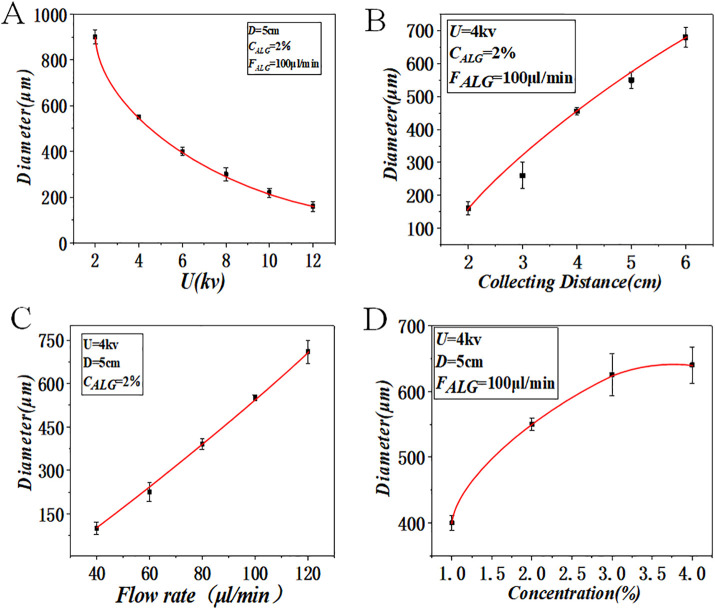
Microsphere size as a function of processing parameters. **(A)** Voltage. **(B)** Collection distance. **(C)** Flow rate. **(D)** Concentration. Collection distance is defined as the vertical distance from the capillary tip to the surface of the CaCl₂ collection bath. Data shown are for microspheres fabricated using a 2% alginate solution unless otherwise specified in the panel. Scale bar = 200 μm (if applicable).

As illustrated in Fig 3A, the mean diameter of the microspheres exhibited a strong inverse correlation with the applied voltage, decreasing from approximately 450 μm at 6 kV to about 180 μm at 12 kV. This reduction is attributed to the increased electrostatic force acting on the forming droplet at the capillary tip, which enhances jet stretching and promotes the formation of smaller, more uniform droplets before gelation.

The collection distance, defined as the vertical span from the tip of the capillary to the surface of the CaCl₂ collection bath, showed a positive influence on microsphere size (Fig 3B). Increasing the distance from 5 cm to 15 cm resulted in a diameter increase of roughly 25%. This is likely due to the longer flight time allowing for greater aerodynamic deformation and solvent evaporation of the alginate jet prior to cross-linking. Similarly, increasing the flow rate from 0.2 mL/h to 0.8 mL/h led to a proportional increase in microsphere diameter (Fig 3C), as a higher volumetric delivery rate provides more material per droplet formation cycle.

The concentration of the sodium alginate precursor solution was a critical determinant of both microsphere morphology and process stability (Fig 3D). At concentrations of 1% and 2%, spherical and highly uniform microspheres were consistently produced. However, at 3% concentration, the increased solution viscosity began to interfere with the electrostatic jet breakup, occasionally leading to slight irregularities in shape and a broader size distribution. Concentrations at or above 4% frequently resulted in unstable jetting and the formation of fibrous structures or large, polydisperse aggregates, rendering them unsuitable for reproducible encapsulation. Therefore, a concentration of 2% was selected as optimal for all subsequent cell encapsulation experiments, providing an ideal balance between gel mechanical stability and processability for monodisperse particle generation.

### Comprehensive Characterization of Microsphere Morphology and Hydrogel Physicochemical Properties

Following the establishment of optimal fabrication parameters, a series of characterization assays were performed to rigorously evaluate the physical and chemical properties of the resultant alginate microspheres.

**Fig 4:**
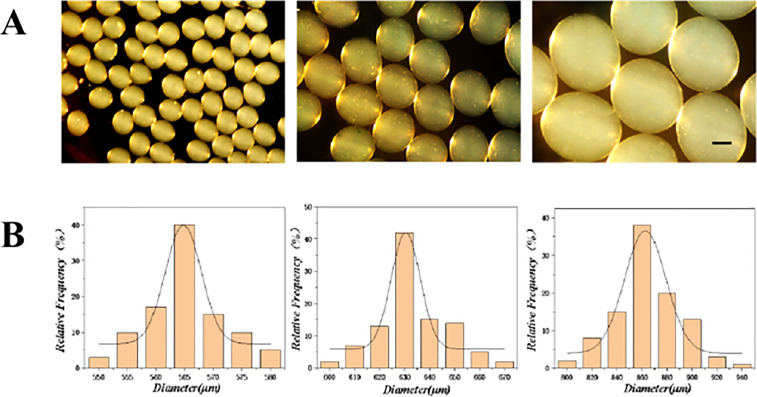
Monodispersity of the prepared microspheres. **(A)** Representative micrographs under different conditions (scale bar: 200 μm). **(B)** Statistical analysis of the monodispersity.

Optical microscopy and image analysis software (ImageJ, NIH) were employed to assess the morphology and size uniformity of the microspheres produced under standardized conditions (2% alginate, 10 kV, 10 cm collection distance, 0.5 mL/h). The microspheres exhibited a high degree of sphericity and monodispersity, with a representative batch showing an average diameter of 320 ± 15 μm (mean ± standard deviation, n > 100) (Fig 4A). The corresponding size distribution histogram confirmed a narrow polydispersity index (Fig 4B), underscoring the reproducibility of our fabrication platform.

**Fig 5:**
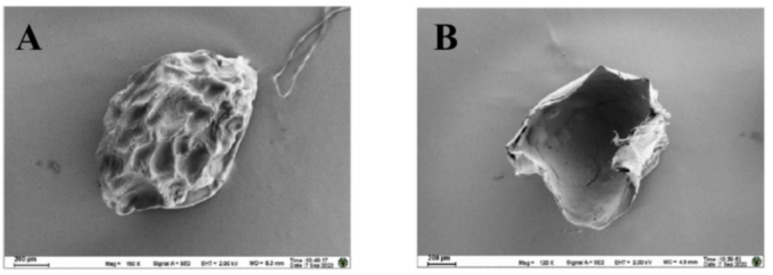
Morphology of the core-shell microspheres. **(A)** Scanning electron micrograph (SEM) of the microspheres. **(B)** Cross-sectional view after freeze-drying. Scale bars: 200 μm.

To elucidate the micro- and nano-scale architecture, microspheres were critical-point dried, sputter-coated with a 10 nm gold-palladium layer, and imaged using a field-emission SEM operated at 5 kV. High-magnification images revealed a smooth and continuous surface topology (Fig 5A). Cross-sectional views of cryo-fractured microspheres, prepared by rapid freezing in liquid nitrogen followed by fracturing, exposed a homogeneous, porous internal structure without discernible phase separation, indicative of a coherent core-shell hydrogel network (Fig 5B).

**Fig 6:**
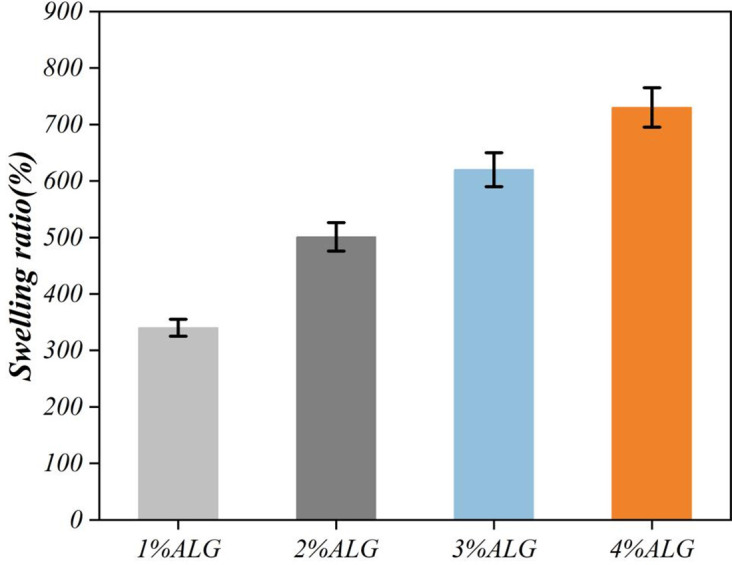
Dissolution rate of sodium alginate as a function of concentration.

The equilibrium swelling ratio, a critical determinant of nutrient and metabolite diffusion, was quantified in phosphate-buffered saline (PBS, pH 7.4) at 37°C. Freeze-dried microspheres of known dry weight (W₀) were immersed in PBS for 48 hours. The surface-adherent liquid was gently removed with filter paper, and the swollen weight (Wₓ) was recorded. The swelling ratio (Q) was calculated as Q = [(Wₓ – W₀)/ W₀] × 100%. As shown in Fig 6, the swelling ratio demonstrated a positive correlation with initial alginate concentration, increasing from approximately 1200% for 1% alginate to over 2000% for 4% alginate, reflecting the increased water uptake capacity of hydrogels with higher polymer content.

**Fig 7:**
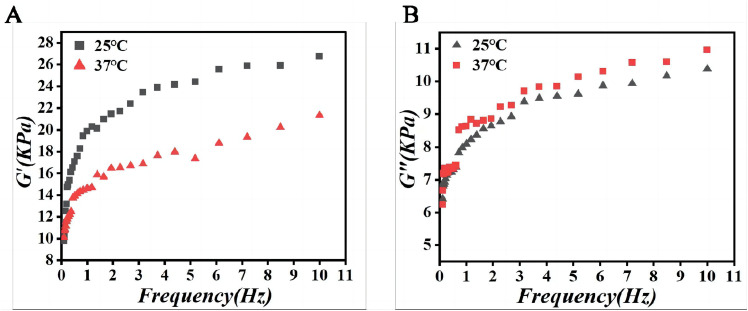
Frequency-dependent viscoelastic behavior of sodium alginate. **(A)** Storage modulus (G’). **(B)** Loss modulus (G“).

The mechanical properties of bulk alginate hydrogel were characterized using a rotational rheometer equipped with a 20 mm diameter parallel-plate geometry. Disc-shaped hydrogel samples (1 mm thickness) were subjected to oscillatory frequency sweeps (0.1 to 10 Hz) at a constant strain of 0.2% and temperatures of 25°C and 37°C. As plotted in Fig 7, both the elastic storage modulus (G′) and the viscous loss modulus (G″) exhibited frequency-dependent behavior across the tested range. G′ consistently remained greater than G″ by an order of magnitude, confirming the solid-like, elastic-dominated character of the cross-linked alginate gel. This stable viscoelastic profile is conducive to maintaining structural integrity under physiological shear stresses while providing a soft microenvironment for encapsulated cells.

### 3.4. In Vitro Biocompatibility and Spatial Organization of Islet Cells within 3D Microspheres

The foundational requirement for any cell encapsulation system is biocompatibility. To rigorously assess this, we performed a quantitative live/dead assay. Microspheres were incubated in direct contact with α-TC6 and β-TC6 cells (co-cultured at a 2:8 ratio) for 24, 48, and 72 hours. At each time point, cells were stained using a solution containing 2 μM Calcein-AM and 4.5 μM propidium iodide (PI) in PBS for 30 minutes at 37°C, protected from light. Calcein-AM, metabolized by esterases in living cells, produces green fluorescence, whereas PI only penetrates the compromised membranes of dead cells, emitting red fluorescence.

**Fig 8:**
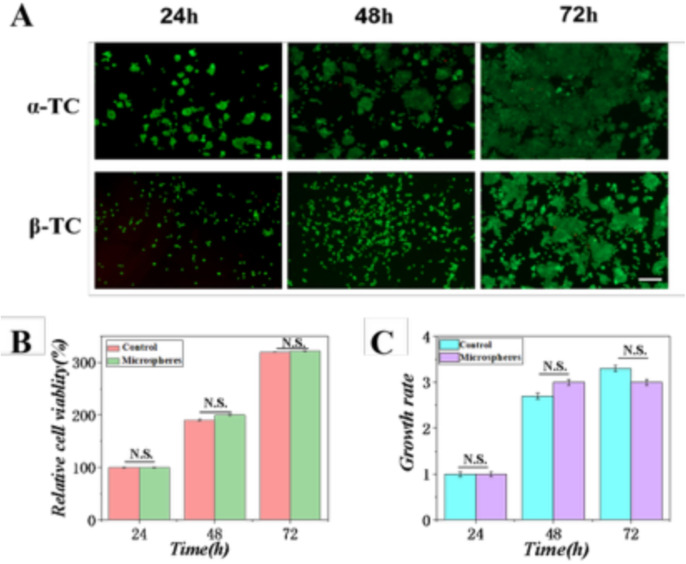
Biocompatibility assessment of sodium alginate hydrogel. **(A)** Live/dead staining of cells cultured with the hydrogel over time (scale bar: 100 μm). **(B)** Cell viability at 24, 48, and 72 hours. **(C)** Cell proliferation rate.

Fluorescence microscopy images (Fig 8A) revealed a predominant and sustained green signal across all time points, with minimal red fluorescence detected. Quantitative image analysis using ImageJ software (thresholding and particle counting) was performed on three independent samples (with five random fields per sample). The calculated cell viability (percentage of Calcein-AM-positive cells relative to total cells) consistently exceeded 95% at 24 h (96.3 ± 1.8%), 48 h (95.7 ± 2.1%), and 72 h (95.1 ± 2.4%), showing no statistically significant difference (p > 0.05, one-way ANOVA) compared to the control group of cells cultured in the absence of microspheres (Fig 8B). This confirms that the alginate microspheres and the fabrication process impose no acute or short-term cytotoxic effects.

Having established biocompatibility, we next visualized the spatial organization of the two islet cell types within the 3D microsphere construct. Prior to encapsulation, α-TC6 and β-TC6 cells were separately labeled with red (CellTracker™ Red CMTPX) and green (CellTracker™ Green CMFDA) fluorescent cytoplasmic dyes, respectively, according to the manufacturer’s protocol. The labeled cells were then mixed in the 2:8 ratio and co-encapsulated. Confocal laser scanning microscopy (CLSM) Z-stack imaging was employed to capture the three-dimensional distribution.

**Fig 9:**
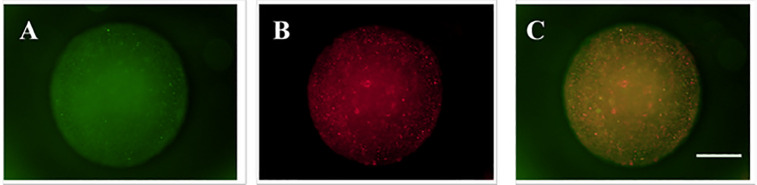
Fluorescence imaging of co-cultured islet cells within microspheres. (A) β-cells (green). (B) α-cells (red). **(C)** Merged image. Scale bar: 200 μm.

As shown in Fig 9, the red fluorescence corresponding to α-TC6 cells (Fig 9B) and the green fluorescence from β-TC6 cells (Fig 9A) were uniformly distributed and intermingled throughout the hydrogel matrix. The merged image (Fig 9C) clearly demonstrates the successful establishment of a 3D co-culture system within a single microsphere, achieving a spatial proximity that mimics the native islet microenvironment and facilitates potential paracrine interactions between the two endocrine cell types.

### 3.5. Preservation of Encapsulated Islet Cell Endocrine Function In Vitro

A pivotal assessment for any encapsulation system is whether the physical barrier imposes functional diffusion limitations that compromise the core physiological activity of the cells—in this case, the regulated secretion of hormones.

**Fig 10:**
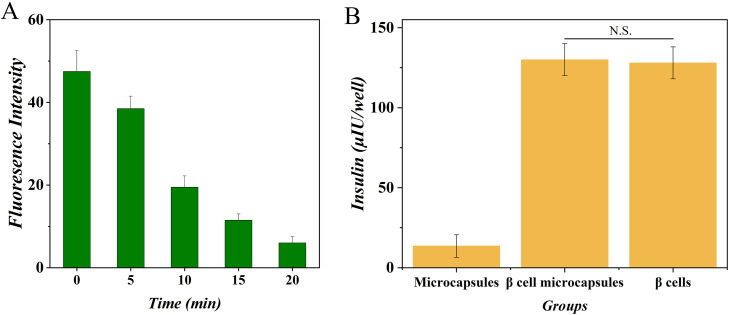
Insulin release profile and microsphere permeability. **(A)** Time-dependent fluorescence intensity of dye diffusion. **(B)** Quantification of insulin secretion under different conditions.

To first establish the baseline diffusional characteristics of the fabricated microspheres, we employed a fluorescence tracer assay. Microspheres were incubated in a PBS solution containing 0.1 mg/mL fluorescein isothiocyanate (FITC)-labeled dextran (average molecular weight: 4 kDa), a molecule comparable in size to insulin (5.8 kDa). The fluorescence intensity inside the microspheres was monitored in real-time using confocal microscopy. As shown in Fig 10A, the fluorescent signal rapidly permeated the microspheres, reaching a uniform distribution plateau within 30 minutes. This confirmed the hydrogel network’s porosity is sufficient for the free and rapid diffusion of small peptides and signaling molecules, a prerequisite for unimpeded hormone exchange.

**Fig 11:**
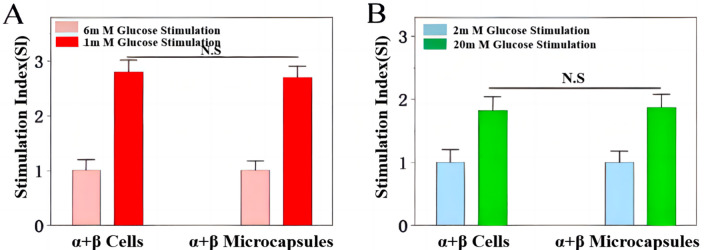
Microsphere impact on islet cell hormone secretion. **(A)** Glucagon secretion. **(B)** Insulin secretion.

We next directly measured the secretory function of encapsulated versus free (unencapsulated) cells. For insulin secretion assays, β-TC6 cells (alone) or the α/β co-culture (2:8 ratio) were either encapsulated or left as free clusters. After a 1-hour pre-incubation in low-glucose Krebs-Ringer Bicarbonate buffer (KRB, 2.8 mM glucose), cells were stimulated for 1 hour with high-glucose KRB (20 mM). The supernatant was collected, and insulin concentration was quantified using a high-sensitivity mouse insulin ELISA kit. The results demonstrated that encapsulation within alginate microspheres did not significantly attenuate the glucose-stimulated insulin secretion (GSIS) response. The secreted insulin levels from encapsulated β-cells or co-cultures were statistically indistinguishable from their free-cell counterparts (p > 0.05) (Fig 10B, Fig 11B).

Parallel experiments were conducted for glucagon secretion. The α/β co-culture systems (encapsulated and free) were first incubated in a standard glucose medium (6 mM), then stimulated with low-glucose KRB (1 mM) for 1 hour. The secreted glucagon was measured using a specific radioimmunoassay. As presented in Fig 11A, the low-glucose-stimulated glucagon release from the encapsulated co-culture was fully preserved, showing no significant difference from the free-cell control group.

### 3.6. Therapeutic Efficacy and Biocompatibility of Cell-Laden Microspheres in a Diabetic Mouse Model

**Fig 12:**
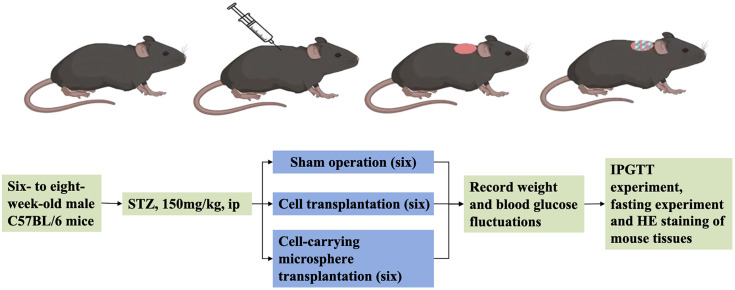
Animal modeling process.

*The ultimate validation of our engineered microsphere system was its performance in a therapeutic in vivo context. Streptozotocin (STZ)-induced diabetic C57BL/6 male mice (blood glucose persistently >16.5 mmol/L one week post-injection) were used as the disease model. Mice were randomly assigned into three experimental cohorts (n = 6 per group): a diabetic sham-surgery control group (DM), a group receiving transplantation of free α and β cells (α+βcells), and the treatment group receiving alginate microsphere-encapsulated α/β cells (α + βMicrocapsules). All grafts contained a total of 2 × 10⁶ cells at a 2:8 α/β ratio and were surgically implanted into the subcutaneous brown adipose tissue depot (* Fig 12 *). Metabolic and physiological parameters were monitored for four weeks post-transplantation.*

**Fig 13:**
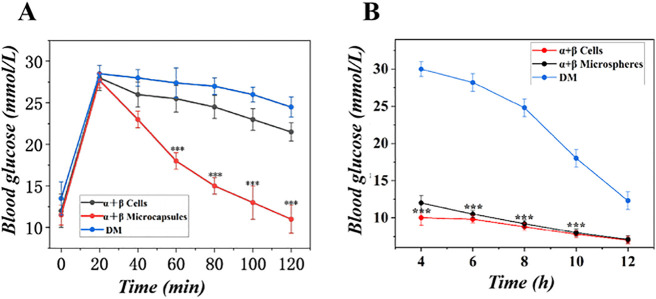
Glucose tolerance and basal metabolic state. **(A)** Intraperitoneal glucose tolerance test (IPGTT) curves. **(B)** Fasting blood glucose levels. (n = 6, ***p < 0.001).

*At week 2 post-op, an intraperitoneal glucose tolerance test (IPGTT) was conducted after a 6-hour fast. Mice were injected with glucose (2 g/kg body weight), and blood glucose was measured via tail-vein sampling at t = 0, 15, 30, 60, and 120 minutes. As shown in* Fig 13A*, the α + βMicrocapsules group exhibited a significantly attenuated glycemic excursion. The area under the curve (AUC) for this group was reduced by approximately 40% compared to the DM group (p < 0.001) and by 30% compared to the α+βcells group (p < 0.01), indicating a markedly improved ability to clear a glucose challenge. Furthermore, weekly measurements of fasting blood glucose (FBG, 6-hour fast) revealed that only the α + βMicrocapsules group achieved and sustained near-normoglycemia (FBG < 11.1 mmol/L) throughout the study period, whereas both the DM and α+βcells groups remained severely hyperglycemic (*Fig 13B*).*

**Fig 14:**
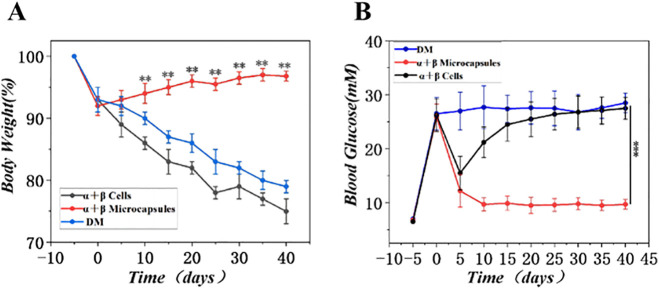
Post-transplantation monitoring of body weight and blood glucose levels in mice. **(A)** Body weight. **(B)** Blood glucose levels. (n = 6, **p < 0.01, ***p < 0.001).

*Diabetes-associated catabolic weight loss was effectively reversed by the microsphere treatment. Prior to transplantation, all STZ-treated mice showed significant weight reduction. Following intervention, the body weight of mice in the α + βMicrocapsules group showed a consistent upward trajectory, increasing by an average of 12.5% from baseline by the end of week 4. In stark contrast, mice in the DM and α+βcells groups continued to lose weight or showed no recovery (*Fig 14A*). This was paralleled by the normalization of random-fed blood glucose levels in the treatment group. As plotted in* Fig 14B*, the α + βMicrocapsules group maintained blood glucose levels significantly lower than the other two groups from week 1 onward (p < 0.001 at all weekly time points), demonstrating a sustained therapeutic effect.*

**Fig 15:**
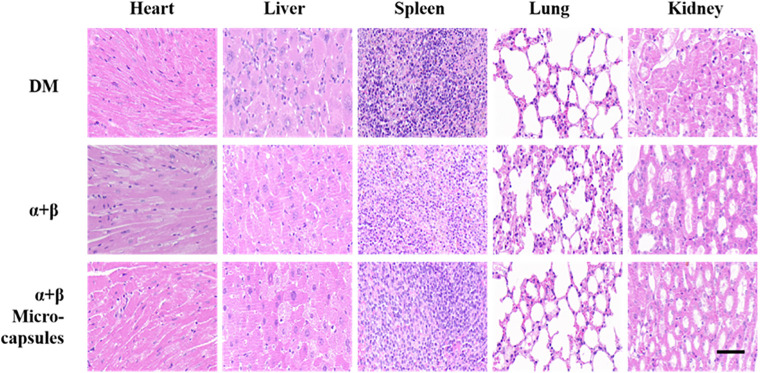
In vivo biocompatibility assessment of the hydrogel post-implantation. Scale bar: 100 μm.

*At the experimental endpoint (week 4), a full necropsy was performed. Histopathological evaluation of major organs—including heart, liver, spleen, lungs, and kidneys—was conducted on hematoxylin and eosin (H&E)-stained paraffin sections. Microscopic examination at 200x magnification revealed no evidence of adverse tissue reactions attributable to the implant or its degradation products. Specifically, there was no significant lymphocytic infiltration, granuloma formation, fibrosis, or abnormal architecture in any of the organs harvested from the α + βMicrocapsules group (* Fig 15 *). The histological profiles were indistinguishable from those of the control groups, providing compelling evidence for the excellent in vivo biocompatibility and safety of our alginate microsphere-based cell delivery system.*

## Discussion

This study successfully demonstrates the preparation of monodisperse sodium alginate microspheres using a microfluidic electrostatic spraying platform. We established that key processing parameters—voltage, collection distance, flow rate, and solution concentration—precisely control the microsphere diameter. Optimal conditions yielded microspheres with uniform size and excellent monodispersity, which is critical for consistent performance in biomedical applications. This level of dimensional control addresses a long-standing challenge in conventional encapsulation methods, such as bulk emulsification or extrusion, which often produce polydisperse populations with unpredictable diffusion kinetics and host responses [16,17].

Comprehensive characterization confirmed the favorable properties of the fabricated microspheres. The swelling behavior and viscoelastic properties of sodium alginate, as illustrated by the frequency-dependent storage (G’) and loss (G“) moduli, are crucial for their function. These characteristics ensure efficient nutrient/waste exchange and provide a mechanically supportive microenvironment that mimics the native extracellular matrix, thereby promoting the viability and functionality of encapsulated cells. The observed G’ consistently exceeding G” by an order of magnitude confirms the elastic-dominant nature of the hydrogel, a feature previously shown to be essential for maintaining structural integrity under physiological shear stresses while providing a compliant niche for encapsulated cells [16,19].

In co-culture experiments with sodium alginate, the material showed no significant impact on normal cellular activities. The permeation and diffusion of sodium alginate spheres were tested using fluorescent proteins, and the release of insulin and glucagon was measured, confirming the material’s compatibility with cell culture. Critically, the unimpaired glucose-stimulated insulin secretion (GSIS) from encapsulated β-cells (Fig 10B, 11B) demonstrates that the alginate barrier, while providing immunoprotection, does not create a functional diffusion barrier for hormone release—a concern previously raised in studies using denser hydrogel formulations [15].Sodium alginate’s biocompatibility makes it a promising material for applications in organ transplantation and other biomedical fields. Its hydrogel form provides a porous structure that facilitates the free exchange of small molecules while sequestering immune cells, thereby mimicking the natural extracellular matrix environment. This 3D structure supports nutrient and waste exchange, enhancing cell survival and function.

The study also highlighted the importance of microsphere shape in organoid construction, opting for spherical sodium alginate microspheres to emulate the islet’s natural environment and promote cell function and nutrient exchange. Despite these advancements, the study acknowledges the need for further improvement and exploration, particularly regarding the impact of microsphere parameters on islet cell growth and the molecular mechanisms underlying cell metabolism within the microspheres.

Insulin and glucagon are pivotal in glucose homeostasis, with insulin lowering blood sugar levels by promoting glucose uptake by tissues and glucagon raising them by stimulating glucose and fatty acid release from the liver. Islet transplantation offers a definitive treatment for diabetes, yet it faces challenges such as donor shortages, post-transplant islet cell survival, and the requirement for long-term immunosuppressive therapy. Encapsulation of islet cells within biocompatible materials like sodium alginate has been explored to enhance cell survival and function.

A key innovation of this study is the deliberate co-encapsulation of α- and β-cells. Most prior encapsulation strategies have focused exclusively on β-cells [13,15], overlooking the essential paracrine crosstalk between α- and β-cells that maintains minute-to-minute glucose homeostasis in vivo. While recent advances in zwitterionic modification of alginate have shown promise in mitigating foreign body responses [22,23], these studies have largely employed single-cell systems. Our co-culture approach, by preserving both insulin and glucagon secretory capacity (Fig 11), more faithfully recapitulates native islet physiology and may offer superior protection against hypoglycemia—a dangerous complication associated with β-cell-only grafts [5,9].

The microfluidic system in this study allowed precise control over microsphere diameter by adjusting parameters such as biomaterial hydrogel concentration, DC voltage, and the distance between the liquid outlet and the collecting liquid surface. This resulted in uniformly sized spherical droplets that solidified upon contact with liquid calcium chloride. The biocompatibility of the hydrogel was confirmed through co-culture with cells, and the microspheres’ permeability to substances of similar molecular weight to insulin was demonstrated. Insulin and glucagon release experiments indicated that the microspheres did not impede the cells’ functionality.

In vivo experiments involved implanting microspheres containing islet α-TC cells and β-TC cells into diabetic mice. Glucose tolerance tests showed improved glucose response in mice with transplanted microspheres, suggesting effective blood sugar regulation. Monitoring of body weight changes post-implantation indicated an alleviation of diabetes symptoms in the treated group. Histological examination of internal organs further confirmed the safety and biocompatibility of the microspheres. The sustained normoglycemia achieved in the α + βMicrocapsules group for four weeks (Fig 14B) compares favorably with previous reports of alginate-encapsulated islet transplants, where graft function often declines within two to three weeks due to fibrotic overgrowth [18,20]. We attribute this improved durability to the combination of uniform microsphere geometry (minimizing “dead spots” for diffusion) and the favorable immune-privileged properties of the subcutaneous brown fat transplantation site, which has been suggested to offer better vascularization than traditional intraperitoneal sites [18].

The study’s innovation lies in the use of two types of islet cells to simulate the natural composition and dual-function glucose regulation of human islets. Sodium alginate was used as an encapsulation material to form microspheres containing α-TC and β-TC cells, which significantly improved common diabetes symptoms in STZ-induced diabetic mice. This approach presents a novel method for constructing islet organoids and holds potential for diabetes drug development.

Despite these findings, the study recognizes limitations, including a small sample size in animal experiments and an unclear mechanism of graft action on blood glucose regulation. Specifically, while we infer immunoprotection from the superior performance of encapsulated versus free cells, direct evidence of immune cell exclusion was not obtained. Furthermore, the durability of graft function beyond four weeks and the potential for late-stage fibrotic responses—a well-documented challenge for alginate-based implants [22,23]—remain to be evaluated. Future research should expand sample sizes, explore the specific mechanisms (e.g., the role of α-cell-derived glucagon in counter-regulation, and the molecular pathways mediating graft-host integration), and extend studies to primate models for higher clinical relevance. Incorporating recent advances in immune-evasive hydrogel chemistries [22–24] into our co-culture system represents a promising direction for further enhancing graft longevity.

## Conclusion

This study focused on the hydrogel-based co-culture of two types of pancreatic islet cells and their applications, in which sodium alginate hydrogels loaded with α-TC cells and β-TC cells were used and their functions were verified in STZ-induced diabetic mice. In vitro, Calcein-AM/PI live/dead staining demonstrated that encapsulation within alginate microspheres preserved cell viability, with survival rates exceeding 95% after 72 hours of co-culture. Hormone secretion assays confirmed that encapsulated cells retained their endocrine function: glucose-stimulated insulin secretion and low-glucose-stimulated glucagon release were both statistically indistinguishable from unencapsulated controls, indicating that the alginate barrier imposed no functional inhibition on the co-cultured islet cells. In vivo, the cell-laden microspheres were transplanted into the subcutaneous brown fat of diabetic mice. Treated animals exhibited significant therapeutic benefits, including a 40% reduction in glucose excursion during IPGTT, sustained near-normoglycemia (fasting blood glucose <11.1 mmol/L) for four weeks, and reversal of diabetes-associated weight loss with a 12.5% increase from baseline. Histopathological examination of major organs (heart, liver, spleen, lungs, kidneys) revealed no evidence of inflammatory infiltration or tissue damage, further confirming the excellent in vivo biocompatibility of the alginate microspheres. Collectively, these findings demonstrate that the sodium alginate microsphere system supports the survival and dual-hormone secretory function of co-cultured α- and β-cells, enables bidirectional blood glucose regulation, and provides effective immunoprotection for the encapsulated cells. This biomimetic platform offers a promising strategy for islet replacement therapy and serves as a physiologically relevant in vitro model for diabetes research, drug discovery, and future clinical applications.

## Supporting information

S1 Data Summary of underlying data. (XLSX)
